# Deletion of B-cell translocation gene 2 (BTG2) alters the responses of glial cells in white matter to chronic cerebral hypoperfusion

**DOI:** 10.1186/s12974-021-02135-w

**Published:** 2021-04-03

**Authors:** Kaoru Suzuki, Mitsuru Shinohara, Yoshihiro Uno, Yoshitaka Tashiro, Ghupurjan Gheni, Miho Yamamoto, Akio Fukumori, Akihiko Shindo, Tomoji Mashimo, Hidekazu Tomimoto, Naoyuki Sato

**Affiliations:** 1 Department of Aging Neurobiology, Center for Development of Advanced Medicine for Dementia, National Center for Geriatrics and Gerontology, 7-430, Morioka, Obu, Aichi 474-8511 Japan; 2 Department of Aging Neurobiology, Graduate School of Medicine, Osaka University, 2-2, Yamadaoka, Suita, Osaka, 565-0871 Japan; 3 Institute of Experimental Animal Sciences, Osaka University Medical School, 2-2 Yamadaoka, Suita, Osaka, 565-0871 Japan; 4 Department of Neurology, Graduate School of Medicine, Mie University, 174, Edobashi 2-chome, Tsu, Mie 514-8507 Japan

**Keywords:** BCAS, Chronic hypoperfusion, White matter lesion, Astrocytes, Mac2-positive cells

## Abstract

**Background:**

Subcortical ischemic vascular dementia, one of the major subtypes of vascular dementia, is characterized by lacunar infarcts and white matter lesions caused by chronic cerebral hypoperfusion. In this study, we used a mouse model of bilateral common carotid artery stenosis (BCAS) to investigate the role of B-cell translocation gene 2 (BTG2), an antiproliferation gene, in the white matter glial response to chronic cerebral hypoperfusion.

**Methods:**

*Btg2*^*−/−*^ mice and littermate wild-type control mice underwent BCAS or sham operation. Behavior phenotypes were assessed by open-field test and Morris water maze test. Brain tissues were analyzed for the degree of white matter lesions and glial changes. To further confirm the effects of *Btg2* deletion on proliferation of glial cells in vitro, BrdU incorporation was investigated in mixed glial cells derived from wild-type and *Btg2*^*−/−*^ mice.

**Results:**

Relative to wild-type mice with or without BCAS, BCAS-treated *Btg2*^*−/−*^ mice exhibited elevated spontaneous locomotor activity and poorer spatial learning ability. Although the severities of white matter lesions did not significantly differ between wild-type and *Btg2*^*−/−*^ mice after BCAS, the immunoreactivities of GFAP, a marker of astrocytes, and Mac2, a marker of activated microglia and macrophages, in the white matter of the optic tract were higher in BCAS-treated *Btg2*^*−/−*^ mice than in BCAS-treated wild-type mice. The expression level of *Gfap* was also significantly elevated in BCAS-treated *Btg2*^*−/−*^ mice. In vitro analysis showed that BrdU incorporation in mixed glial cells in response to inflammatory stimulation associated with cerebral hypoperfusion was higher in *Btg2*^*−/−*^ mice than in wild-type mice.

**Conclusion:**

BTG2 negatively regulates glial cell proliferation in response to cerebral hypoperfusion, resulting in behavioral changes.

**Supplementary Information:**

The online version contains supplementary material available at 10.1186/s12974-021-02135-w.

## Background

Subcortical ischemic vascular dementia, one of the major subtypes of vascular dementia, is characterized by lacunar infarcts and white matter lesions caused by chronic cerebral hypoperfusion [1–3]. Chronic cerebral hypoperfusion is modeled in rodents by bilateral common carotid artery stenosis (BCAS), in which micro-coils are placed bilaterally on the common carotid arteries [4–6]. BCAS model mice exhibit apparent glial activation in addition to white matter lesions, providing important insight into the mechanism of subcortical ischemic vascular dementia [4]. Brain inflammation, defined by glial activation, has attracted a great deal of attention in stroke research [7–10]. Notably in this regard, proliferation of glial cells, including astrocytes, is observed after ischemic injury in animal models [11–13].

B-cell translocation gene 2 (BTG2, also called PC3/Tis21) was identified as an anti-proliferative protein regulated by p53 activation [14] and was initially described as an immediate early gene induced by growth factors and tumor promoters in PC12 and Swiss 3T3 cells [15, 16]. BTG2 belongs to the BTG/TOB family, whose members regulate cell-cycle progression, apoptosis, and differentiation [17, 18]. In particular, BTG2 is involved in several biological processes including cell-cycle progression [14], induction of cellular senescence [19], cellular differentiation including neurogenesis [20], hematopoiesis [21–24], the genotoxic stress response [14, 25, 26], and integrated stress responses such as the response against oxidative stress [18, 27]. *Btg2* is broadly expressed in multiple organs, including the kidney, lung, prostate, pancreas, thymus, skeletal muscle, and central nervous system [18, 28–30]. In the mouse brain, *Btg2* is highly expressed in astrocytes, microglia, and endothelial cells (https://www.brainrnaseq.org). Notably, *Btg2* mRNA is induced in the brain response to hypoxia–ischemia [31] and in the brains of AD model mice with obesity-linked diabetes but not in those of AD model mice or mice with obesity-linked diabetes [32]. Moreover, *Btg2* is also upregulated during chronic cerebral hypoperfusion in BCAS-treated mice [33]. However, the roles of BTG2 in chronic cerebral hypoperfusion remain unclear. To answer this question, in this study, we generated *Btg2*^*−/−*^ mice, performed BCAS surgery, and analyzed locomotor activity, cognitive behavior, white matter lesions, and glial cells in the white matter.

## Experimental procedures

### Animals

All mice shared the same genetic background (C57BL/6J). The mice were group housed without enrichment structures in a specific pathogen-free environment in ventilated cages and were used in the experiments according to the Guideline for the Care and Use of Laboratory Animals of our research facilities. Several strains of *Btg2*^*−*/*−*^ mice were produced by electroporation using the CRISPR/Cas9 system [34] at the Institute of Experimental Animal Sciences, Graduate School of Medicine, Osaka University. Briefly, the following reagents were purchased: Cas9 mRNA, T7-NLS-hCas9-polyA (RIKEN BRC #RDB13130), and guide RNA (gRNA). To design the gRNA sequence (5′-ACATGCTCCCGGAGATCGCC-3′), a software tool (http://crispr.mit.edu/) for predicting unique target sites in the mouse genome was used. The specificity of CRISPR/Cas9-mediated DNA cleavage is conferred by the 20-base sequence of the single-guide RNA (sgRNA) targeting the chromosomal mouse *Btg2* genomic sequence. The CCG trinucleotide, the protospacer-adjacent motif (PAM), which is recognized by Cas9, resides 16 bases downstream from the start codon of the mouse *Btg2* genomic sequence. Pronuclear-stage mouse embryos were prepared by thawing frozen embryos (CLEA Japan Inc., Japan). For electroporation, 50–100 embryos at 1 h after thawing were placed into a chamber with 40 μl of serum-free media (Opti-MEM, Thermo Fisher Scientific, USA) containing 400 ng/μl Cas9 mRNA and 200 ng/μl gRNA and then electroporated with a 5-mm gap electrode (CUY505P5 or CUY520P5 Nepa Gene, Chiba, Japan) in a NEPA21 Super Electroporator (Nepa Gene, Chiba, Japan). After introduction of Cas9 and gRNA, mouse embryos were transferred to the oviduct of a female surrogate anesthetized with sevoflurane (Mylan Pfizer Japan Inc.). We obtained a line of *Btg2*^*−/−*^ mice with an 11-bp deletion of the *Btg2* genomic sequence. Genotype was confirmed by PCR using the following primers: forward: 5′-CTCCCCGAGTGGTATGAAAG-3′, reverse: 5′-TCAAGGTTTTCAGTAGGGCG-3′. When PCR products were separated by 6% acrylamide gel electrophoresis, two products were detected: 220 bp, indicating the *Btg2* deletion, and 231 bp, indicating the wild type. Sanger sequencing of PCR products from wild-type and *Btg2*^*−/−*^ mice was performed using the same set of primers (Japan GENEWIZ, Saitama, Japan). To exclude off-target effects, heterozygous *Btg2*^+/−^ mice were backcrossed for six generations to wild-type C57BL/6J mice.

### BCAS surgery

Surgical procedures for BCAS were described previously [4]. Briefly, mice were habituated in the surgery room with free access to food and water for 3–5 days before surgery at 26 °C under a 12-h/12-h light/dark cycle. Nine- to eleven-week-old wild-type mice and *Btg2*^*−/−*^ littermates (body weight, 22–28 g) underwent BCAS or sham operation. Before surgery, mice were intraperitoneally injected with a combination of three anesthetics: 0.3 mg/kg medetomidine hydrochloride (Nippon Zenyaku Kogyo, Fukushima, Japan), 4.0 mg/kg midazolam (Astellas, Tokyo, Japan), and 5.0 mg/kg butorphanol tartrate (Meiji Seika Pharma, Gifu, Japan). For sham-operated mice, the common carotid artery (CAA) was exposed and carefully freed from its sheath. For BCAS-treated mice, an 0.18-mm inner diameter micro-coil (Samini, Shizuoka, Japan) was then attached bilaterally to the CAA. After surgery, 3.0 mg/kg atipamezole hydrochloride (Nippon Zenyaku Kogyo), an antagonist of medetomidine hydrochloride, was injected intraperitoneally to promote safe awakening. To maintain body temperature after surgery, the mice were warmed with a heating pad at 37 °C.

### Behavioral tests

To assess the effect of *Btg2* deletion on the behavior of the BCAS model, we performed the open-field and Morris water maze tests. One month after the surgery, the open-field test was performed: mice were placed in a cubical plastic box (30 × 30 × 30 cm^3^, width × length × height) and allowed to roam freely for 15 min. One and a half months after the surgery, the Morris water maze test was performed to test spatial learning and memory, as previously described [35, 36]. The test was conducted in a circular pool filled with water adjusted to ambient temperature. For hidden platform training, a transparent platform was submerged 1.5 cm below water level. The pool was located in a test room in which there were many external cues. On day 1, a preparative session was carried out in which mice were given 90 s for free swimming without the platform, followed by two training trials (1 session) with the platform. From day 2 to day 5, four training trials (two sessions) were performed each day. During each trial, the mice were released from four pseudo-randomly assigned starting points and allowed to swim for 90 s. The inter-trial interval was approximately 10 min, and the intersession interval was 2 h. After mounting the platform, the mice were allowed to remain there for 10 s and were then placed in a holding cage with a heating lamp until the start of the next trial. If a mouse failed to find the platform, it was guided to the platform and allowed to rest on the platform for 10 s. The probe test was performed 24 h after the last hidden platform training. In the probe test, the hidden platform was removed, and the mouse was released from the opposite quadrant and allowed to swim freely for 60 s. In the visible platform test performed after the last probe test on the same day, the platform was elevated above the water surface. The mice underwent three trials lasting 90 s. All experiments were conducted at approximately the same time each day. An overhead camera was used to track the movement of mice in both the open-field and Morris water maze tests, and the video was analyzed using the ANY-maze software (Muromachi Kikai, Tokyo, Japan).

### Tissue collection and preparation

Seven weeks after surgery, mice were anesthetized with isoflurane (Fujifilm Wako Pure Chemicals, Osaka, Japan) and perfused transcardially with phosphate-buffered saline (PBS) plus complete protease inhibitor cocktail (Roche Diagnostics, Basel, Switzerland). After perfusion, the brain was removed from the skull and divided along the sagittal plane. The right hemisphere was divided into the cortex plus hippocampal areas and other areas, frozen in liquid nitrogen, and stored at − 80 °C until the biochemical analysis. The left hemisphere was fixed with 4% paraformaldehyde/PBS overnight and embedded in paraffin for histological analysis. For biochemical analysis, tissues were pulverized in a BioPulverizer (BioSpec Products, Bartlesville, OK, USA) that had been prechilled on dry ice, and then stored at − 80 °C.

### Quantitative real-time PCR

Total RNA was purified from pulverized samples using RNeasy Lipid Tissue Mini kit (QIAGEN, Venlo, Netherlands), eluted in nuclease-free water, and stored at − 80 °C. Reverse transcription was performed using ReverTra Ace qPCR RT Master Mix with gDNA remover (TOYOBO, Osaka, Japan). Real-time PCR was conducted with Luna Universal qPCR Master Mix (New England Biolabs, Ipswich, MA, USA) on an i1000 thermal cycler (Bio-Rad Laboratories, Hercules, CA, USA) to detect mRNA levels of *Btg2*, *Btg1*, *Btg3*, *Btg4*, *Tob1*, *Tob2*, *Gfap*, *Cd11b*, *Trem2*, *Dap12*, *Cd45*, *Cd68*, *F4/80*, *Cd14*, and *β-actin*. The following primers were used: *Btg2*, forward CGGGAAGAGAACCGACATGC and reverse 5′-GCCCTACTGAAAACCTTGAGTC-3′; *Btg1*, forward 5′-CCACCATGATAGGCGAGATCG-3′ and reverse 5′-TGCGAATACAACGGTAACCTG-3′; *Btg3*, forward 5′-AAGAACGAAATTGCGGCTGTT-3′ and reverse 5′-CATCGGGATCAACTCTCTGAAAC-3′; *Btg4*, forward 5′-TTCCAAGGGGCAGGCTTTTAG-3′ and reverse 5′-ACCTCATAGGGATCGACCCATA-3′; *Tob1*, forward 5′-ATGCAGCTTGAAATCCAAGTAGC-3′ and reverse 5′-AGGATACCAGTGCCCTTCATATT-3′; *Tob2*, forward 5′-GCTAGAGCGGCTTCTGAGAAA-3′ and reverse 5′-ACCACAGGGTCTACCACTTCC-3′; *Gfap*, forward 5′-CGGAGACGCATCACCTCTG-3′ and reverse 5′-AGGGAGTGGAGGAGTCATTCG-3′; *Cd11b*, forward 5′-GCATGTCAAGAACAAGTA-3′ and reverse 5′-CTAAAGCCAGGTCATAAG-3′; *Trem2*, forward 5′-TGCTGGCAAAGGAAAGGTG-3′ and reverse 5′-GTTGAGGGCTTGGGACAGG-3′; *Dap12*, forward 5′-GTTGACTCTGCTGATTGCCCT-3′ and reverse 5′-CCCTTCCGCTGTCCCTTGA-3′; *Cd45*, forward 5′-GCCCAAACAAATTACACAT-3′ and reverse 5′-TTAGGCGTTTCTGGAATC-3′; *Cd68* forward 5′-CCAATTCAGGGTGGAAGA-3′ and reverse primer 5′-TTGCATTTCCACAGCAGAAG-3′; *F4/80*, forward 5′-ATGGACAAACCAACTTTCAAGGC-3′ and reverse 5′-GCAGACTGAGTTAGGACCACAA-3′; *Cd14*, forward 5′-CTCTGTCCTTAAAGCGGCTTAC-3′ and reverse 5′-GTTGCGGAGGTTCAAGATGTT-3′; and *β-actin*, forward 5′-AGTGTGACGTTGACATCCGTA-3′ and reverse 5′-GCCAGAGCAGTAATCTCCTTC-3′ (Integrated DNA Technologies, Coralville, IA, USA). All primer sequences were obtained from PrimerBank (https://pga.mgh.harvard.edu/primerbank) except those for *F4/80* and *Cd14* [37], *Trem2* and *Dap12* [38], and *Cd11b* and *Cd45* [39]. Relative expression levels were quantified and analyzed using the iCycler iQ software (Bio-Rad). Relative mRNA levels were calculated using the ΔΔCt method, with *β-actin* serving as an internal control for each specific gene amplification reaction.

### ELISA assay

Protein levels of glial fibrillary acidic protein (GFAP) and CD11b in the sham/BCAS-treated wild-type and *Btg2*^*−/−*^ mice brain were determined by ELISA as previously described [38]. In brief, pulverized brain samples were homogenized on ice using a Polytron homogenizer (Kinematica, Luzon, Switzerland) in ice-cold RIPA lysis buffer (10 ml/g of wet weight; MilliporeSigma, Burlington, MA, USA) containing 0.1% SDS and complete protease inhibitor cocktail (Roche). After centrifugation at 100,000*g* for 1 h at 4 °C, the supernatant was aliquoted and analyzed by ELISA with the appropriate sets of antibodies. Colorimetric quantification was performed on an iMark plate reader (Bio-Rad) after incubation with horseradish peroxidase (HRP)-linked Avidin-D (Vector Laboratories, Burlingame, CA, USA) or anti-rabbit IgG (H+L), HRP conjugate (Promega, Madison, WI, USA) and 3,3′,5,5′-tetramethylbenzidine substrate (Sigma Aldrich, St. Louis, MO, USA). Protein concentrations in each sample were performed using the Protein Assay BCA kit (Fujifilm Wako Pure Chemical).

### Histological preparation

Paraffin-embedded brain samples were sectioned at 5-μm thickness by microtome (SM2000R, Leica Microsystems, Wetzlar, Germany). Four non-adjacent sections including the hippocampal region were mounted per slide on MAS (Matsunami Adhesive Slide)-coated glass slides (Matsunami Glass Ind., Osaka, Japan). These tissue samples were kept at room temperature prior to histological examination.

### Klüver–Barrera staining

Paraffin-embedded sections were deparaffinized, hydrated, and immersed in Luxol Fast Blue solution (Muto Chemical, Tokyo, Japan) at 65 °C overnight. Sections were chilled at room temperature, rinsed in 95% ethanol, treated with 0.1% lithium carbonate, rinsed in 70% ethanol, counterstained with 0.1% Cresyl Violet Acetate (Muto Chemical) containing 10% acetic acid, and mounted with Entellan New (Merck Millipore). Sections were analyzed on a BZ-X810 microscope (Keyence, Osaka, Japan). Using images of the white matter area (optic tract and corpus callosum), the severity of white matter lesions was classified into four grades as described by Wakita et al. [40]: normal (grade 0), disarrangement of nerve fibers (grade 1), formation of marked vacuoles (grade 2), and disappearance of myelinated fibers (grade 3). Grading was performed by three individuals in a blinded manner.

### Immunohistochemistry

For immunohistochemistry, brain sections were deparaffinized, hydrated, treated with 0.1% Triton-X/PBS, and treated with 3% H_2_O_2_ solution for inactivation of endogenous peroxidase. After blocking using 1% BSA/PBS, sections were incubated with the following primary antibodies at 4 °C overnight: rabbit anti-GFAP polyclonal (#IS524, Agilent, Santa Clara, CA, USA, 1:1 diluted), rabbit anti-Iba1 polyclonal (#019-19741, Fujifilm Wako Pure Chemicals, 1:100 diluted), and rat anti-Mac2 monoclonal (#CL8942AP, CEDARLANE, Burlington, Canada, 1:1000 diluted). After three washes in PBS, the sections were treated with horseradish peroxidase (HRP)-conjugated secondary antibodies (#W401B, goat anti-rabbit IgG, Promega, WI, USA, or #AP136P, goat anti-rat IgG; Merck Millipore), diluted 1:2500 in 1% BSA/PBS, for 2 h at room temperature. After three washes in PBS, HRP-labeled slides were treated with Impact DAB (Vector Laboratories), a peroxidase substrate. Stained sections were observed on a BZ-X810 microscope. For immunofluorescence observations, sections were incubated at room temperature with anti-rabbit IgG Alexa Fluor 488 (#A11008, Thermo Fisher Scientific, OR, USA) or anti-rat IgG Alexa Fluor 546 (#A11081, Thermo Fisher Scientific) diluted 1:100 in 1% BSA/PBS. Fluorescently labeled sections were mounted in VECTASHIELD Mounting Medium with DAPI (Vector Laboratories) and observed on a LSM700 confocal laser microscope (Zeiss, Oberkochen, Germany).

### Cell culture and BrdU incorporation assay

Mixed glial cell culture was performed as described previously [41, 42]. Briefly, primary mixed glial cells were prepared from the cortex and hippocampus of embryonic 17.5–19.5 wild-type and *Btg2*^*−/−*^ mouse embryos. At 20–25 days in vitro (DIV), glial cells cultured in DMEM/F-12 with 10% fetal bovine serum and 1% penicillin–streptomycin were seeded at the density of 0.5 × 10^5^ cells/well on round cover glasses in 12-well culture plates. For inflammatory stimuli, cells were treated with 100 ng/ml lipopolysaccharide (LPS) (Fujifilm Wako Pure Chemical), 10 U/ml interferon gamma (IFNγ) (R&D Systems, Minneapolis, MN, USA), or 100 ng/ml LPS plus 10 U/ml IFNγ, as previously reported [43]. After 48 h, cells were incubated with 10 μM 5-bromo-2′-deoxyuridine (BrdU) (BrdU Immunohistochemistry Kit, #ab125306, Abcam, Cambridge, UK) for 24 h and simultaneously exposed to inflammatory stimulus or control conditions. To detect BrdU-incorporated cells, fixed cells were treated with 10% H_2_O_2_/MeOH, reacted with trypsin solution and denaturing solution, and incubated with detector antibody (BrdU Immunohistochemistry Kit, Abcam). After incubation with Alexa Fluor 568-conjugated anti-sheep IgG (#ab175713, Abcam), cells were mounted in VECTASHIELD Mounting Medium with DAPI. Cytological observations were performed on an LSM700 microscope.

To identify astrocytes and microglia in mixed glial cells incorporating BrdU, we performed double immunostaining using anti-BrdU antibody and anti-GFAP/anti-Iba1 antibodies. After treatment with 0.3% Triton-X/PBS, microwave was applied in 0.01 M Tris-EDTA (pH 9.0). After blocking with 1% BSA/PBS for 1 h, the fixed cells were incubated with anti-BrdU monoclonal antibody (# 66241-1-lg, ProteinTech, diluted 1:300) and anti-GFAP polyclonal antibody (#IS524, Agilent, diluted 1:1) or anti-Iba1 polyclonal antibody (#019-19741, Fujifilm Wako Pure Chemicals, 1:100 diluted) overnight at 4 °C. Next, the samples were incubated for 2 h at room temperature with secondary antibodies, anti-mouse IgG Alexa Fluor 546-conjugated and anti-rabbit IgG Alexa Fluor 488-conjugated (#A11003 and #A11008, respectively, Thermo Fisher Scientific, diluted 1:100). Specimens were mounted in VECTASHIELD with DAPI and observed under a BZ-X810 microscope.

### Statistical analyses

All statistical analyses were performed using the JMP Pro software (version 14.2, SAS Institute, Cary, USA). In the animal experiments, the effects of each genotype with or without BCAS were analyzed through (1) linear regression models adjusting for sex, followed by Tukey’s HSD test (when both sexes were included), or (2) one-way ANOVA followed by Tukey–Kramer test or Dunnett’s multiple comparison test (when only one sex was included). Morris water maze test results were analyzed by repeated-measures one-way ANOVA followed by Tukey–Kramer test. For gene expression analyses of the BTG/TOB family, Student’s *t*-test was used to compare the two genotypes. In cellular experiments, the effects of genotype with or without treatment were analyzed by one-way ANOVA followed by Tukey–Kramer test. We used standard error of the mean (SEM) for adjusted values by sex in Figs. 3, 4, and 5, and Supplementary Figures 1, 2, and 3, and standard deviation (SD) for non-adjusted values in Figs. 2, 7, and 8, and Supplementary Figures 4 and 5. *p*-values less than 0.05 were considered significant. The individual effect of *Btg2* deletion or BCAS treatment was analyzed by two- or three-way ANOVA (Supplementary Table 1).

## Results

### Development of *Btg2*^*−/−*^ mice

**Fig. 1:**
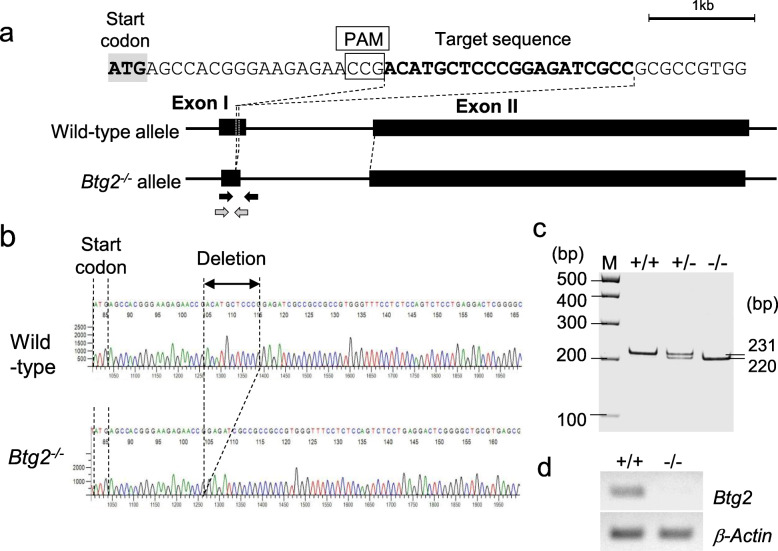
Development of *Btg2*^*−/−*^ mice by genome editing. **a** Target sequence is shown. Black arrows show the primer pair used for genotyping, and white arrows show the primer pairs used for real-time PCR. PAM, protospacer-adjacent motif. **b** Genomic sequence of wild-type mice (upper) and *Btg2*^*−/−*^ mice (bottom). Eleven bases were deleted from exon I of the mouse *Btg2* gene. **c** Genotyping pattern of wild-type (+/+), heterozygous deletion (+/−), and homozygous deletion (*−*/*−*) of *Btg2*, separated on a 6% acrylamide gel. **d** RT-PCR products of total brain RNA from wild-type (+/+) and homozygous deletion of *Btg2* (*−*/*−*), separated on a 2% agarose gel. β-actin was used as a loading control

**Fig. 2:**
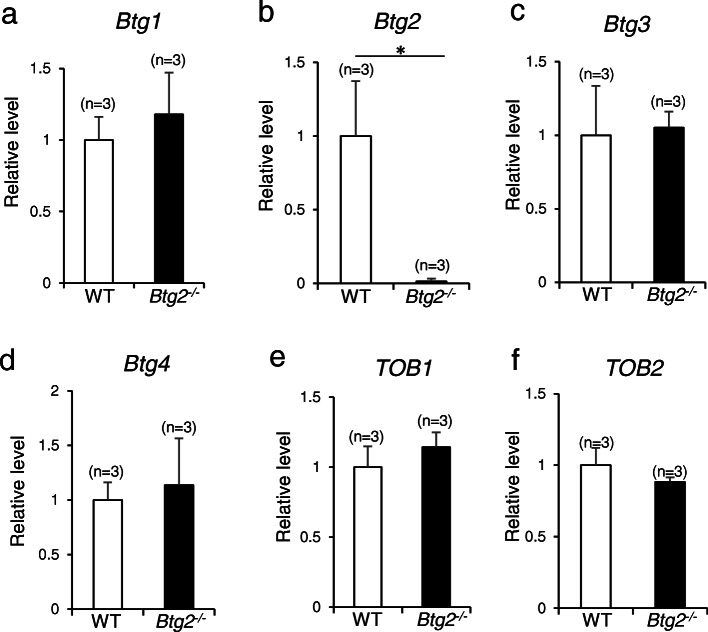
mRNA levels of *Btg*/*Tob* family members **a** *Btg1*, **b** *Btg2*, **c** *Btg3*, **d** *Btg4*, **e** *Tob1*, and **f** *Tob2*, in the brains of female wild-type and *Btg2*^*−/−*^ mice (*n* = 3/group). Means ± SD; *p < 0.05, Student’s *t*-test

To assess the role of *Btg2* in chronic cerebral hypoperfusion, we generated *Btg2*^*−/−*^ mice by genome editing (Fig. 1a–c). RT-PCR revealed that *Btg2* mRNA was absent from *Btg2*^*−/−*^ mice (Fig. 1d). Because BTG2 belongs to the BTG/TOB family, we asked whether the expression of other members of the BTG/TOB family was upregulated by a compensatory mechanism in *Btg2*^*−/−*^ mice. However, expression levels of other members of the BTG/TOB family were not upregulated in *Btg2*^*−/−*^ mice (Fig. 2), suggesting deletion of *Btg2* is not compensated by upregulation of other family members.

### Behavioral characteristics of BCAS-treated *Btg2*^*−/−*^ mice

**Fig. 3:**
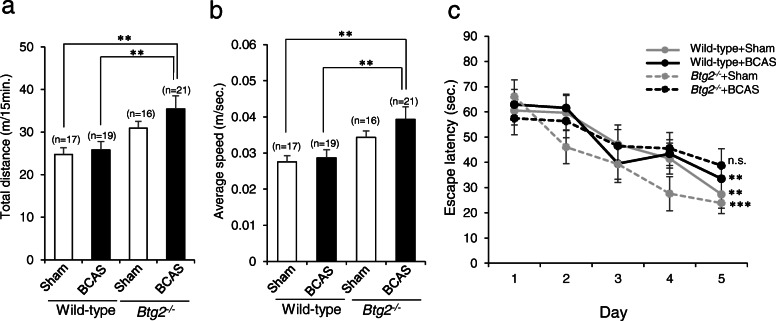
Behavioral changes of sham/BCAS-treated wild-type and *Btg2*^*−/−*^ mice. **a**, **b** Total distance traveled (**a**) and average speed (**b**) in the open-field test. Data are adjusted means ± SEM. **p < 0.01, as determined by Tukey’s HSD test. **c** Average escape latency in each day during the 5-day training trial for the Morris water maze test. Data are adjusted means ± SEM. **p < 0.01 and ***p < 0.001 compared with the scores from day 1 and day 5, as determined by repeated-measures one-way ANOVA followed by Tukey’s HSD test. n.s., not significant

We treated *Btg2*^*−/−*^ mice and littermate wild-type mice with or without BCAS. One month after the surgery, behavioral changes were initially assessed by the open-field test. Total distance traveled (Fig. 3a) and average speed (Fig. 3b) were significantly higher in BCAS-treated *Btg2*^*−/−*^ mice (*p* < 0.01). The ratio of travel distance in the outer or inner part of the open field to total field, which is an index of anxiety or exploratory behavior, respectively, did not significantly differ among groups (Supplementary Fig. 1).

We then examined spatial learning ability using the Morris water maze test; the experiment consisted of 5-day training trials followed by one probe test. We observed no significant difference in the results of the visible platform test (data not shown). Sham-operated wild-type, BCAS-treated wild-type, and sham-operated *Btg2*^*−/−*^ groups exhibited a significant reduction in escape latency on the fifth day relative to the first day (*p* < 0.01, *p* < 0.01, and *p* < 0.001, respectively, Fig. 3c). By contrast, in the BCAS-treated *Btg2*^*−/−*^ group, we observed no such reduction in escape latency (*p* = 0.3306, Fig. 3c), suggesting that the spatial learning ability of BCAS-treated *Btg2*^*−/−*^ mice was impaired relative to other mice. In the probe test, we observed no significant difference among groups in the time spent in the target quadrant (Supplementary Fig. 2).

### Histological observations of white matter in BCAS-treated *Btg2*^*−/−*^ mice

**Fig. 4:**
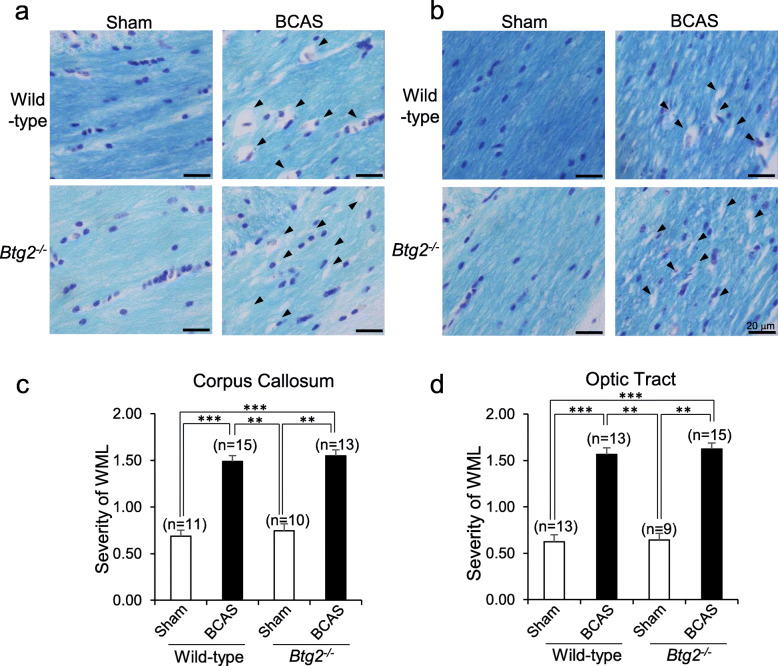
Histological observations of white matter in sham/BCAS-treated wild-type and *Btg2*^*−/−*^ mice. **a**, **b** Corpus callosum (**a**) and optic tract (**b**) subjected to Klüver–Barrera staining. Black arrowheads show vacuolation in myelin fibers. Scale bar = 20 μm. **c**, **d** Severity of white matter lesion (WML) in the corpus callosum (**c**) and optic tract (**d**). All mice were male. Data are adjusted means ± SEM. **p < 0.01 and ***p < 0.001, as determined by Tukey’s HSD test

To assess the effect of *Btg2* deletion on white matter lesions after BCAS treatment, we performed histological examinations of white matter by Klüver–Barrera staining following behavioral assessments. The appearance of myelin fibers in the white matter (corpus callosum and optic tract) was graded for the severities of lesions as described by Wakita et al. [40]. Although the severities of lesions in the corpus callosum and optic tract were significantly increased by BCAS treatment, we observed no significant difference between wild-type and *Btg2*^*−/−*^ mice with or without BCAS (Fig. 4).

### Assessment of glial cell markers in BCAS-treated *Btg2*^*−/−*^ mice

**Fig. 5:**
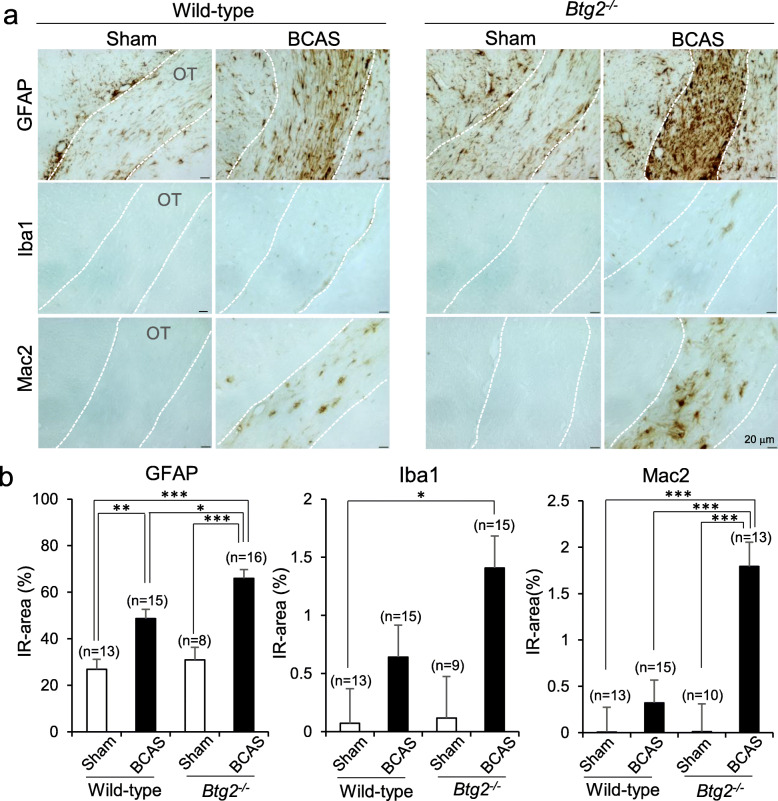
Immunohistochemical observations of glia cells in the optic tract (OT) of sham/BCAS-treated wild-type and *Btg2*^*−/−*^ mice. **a** Staining for GFAP (upper), Iba1 (middle), and Mac2 (lower). Scale bar = 20 μm. OT is inside the white dotted lines. **b** Immunoreactive (IR) areas were compared among groups after adjusting for sex. Data are adjusted means ± SEM. *p < 0.05, **p < 0.01, and ***p < 0.001, as determined by Tukey’s HSD test

**Fig. 6:**
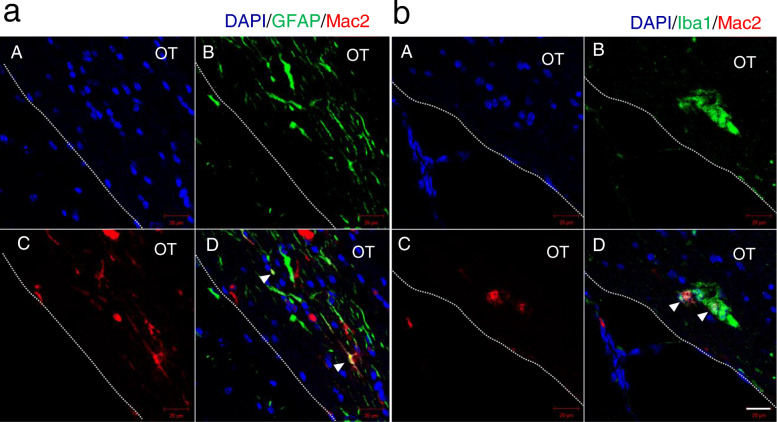
Co-localization of Mac2 and GFAP immunoreactivities (**a**) and Mac2 and Iba1 immunoreactivities (**b**) in the optic tract (OT) of BCAS-treated *Btg2*^*−/−*^ mice. GFAP- and Iba1-positive cells are shown with green fluorescence, and Mac2-positive cells are shown with red fluorescence. White arrowheads show co-localization of Mac2 and GFAP (**a**) or Iba1 immunoreactivities (**b**). Blue fluorescence indicates DAPI-stained cell nuclei. The OT is to the right of the dotted line. Scale bar = 20 μm

To assess the effects of *Btg2* deletion on the response of glial cells to BCAS, we performed immunohistochemical analysis using anti-GFAP, anti-Iba1, and anti-Mac2 antibodies (Fig. 5). The GFAP-immunoreactive area in the optic tract was significantly increased by BCAS. Moreover, BCAS treatment also increased the GFAP- and Mac2-immunoreactive areas in *Btg2*^*−/−*^ mice relative to wild-type mice (Fig. 5b). Iba1-immunoreactive areas were larger in BCAS-treated *Btg2*^*−/−*^ mice than in sham-operated wild-type mice. In the corpus callosum, we observed similar trends in GFAP and Iba1 immunoreactivities in the optic tract following BCAS treatment, but no significant difference in the GFAP- and Mac2-immunoreactive areas in mice harboring the *Btg2* deletion (Supplementary Fig. 3). To investigate the relationship between Mac2- and GFAP- or Iba1-positive cells, we performed double-staining (Fig. 6). Although most Mac2-positive cells did not contain GFAP or Iba1 immunoreactivities, co-localization of Mac2 and GFAP or Iba1 immunoreactivities was observed in some cells (Fig. 6a and b, respectively) (white arrowheads).

**Fig. 7:**
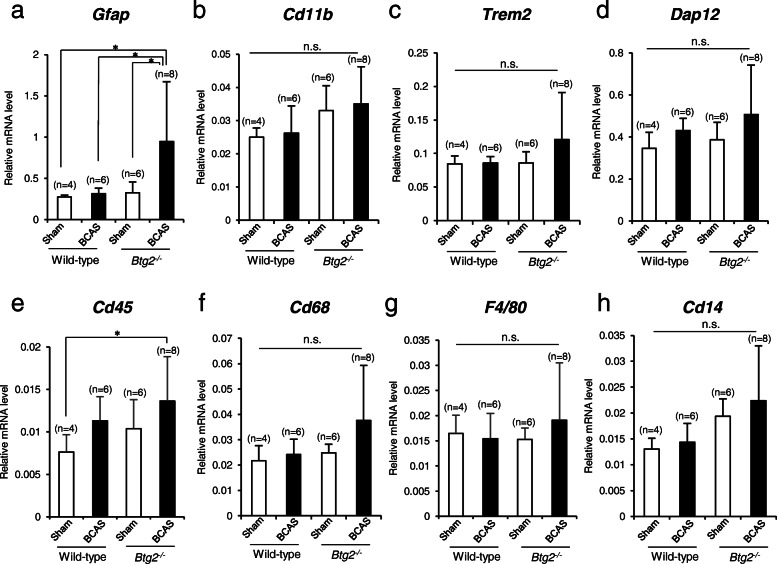
Relative mRNA levels of cell-specific markers in brains of sham/BCAS-treated wild-type and *Btg2*^*−/−*^ mice. mRNA levels of **a** *Gfap*, an astrocyte marker; **b** *Cd11b*, a microglia, monocyte, and macrophage marker; **c** *Trem2* and **d** *Dap12*, disease-associated microglia (DAM) markers; **e** *Cd45*, a pan-hematopoietic marker; **f** *Cd68*, a macrophage marker; **g** *F4/80*, a mature macrophage marker; and **h** *Cd14*, a monocyte and macrophage marker, were compared among groups. All mice were male. Data are means ± SD. *p < 0.05, as determined by Dunnett’s multiple comparison test. n.s., not significant

To analyze the changes in these glial cells by other means, we performed real-time PCR to quantify mRNA levels of the following representative markers: *Gfap* (astrocytes), *Cd11b* (microglia, monocytes, and macrophages), *Trem2* and *Dap12* (disease-associated microglia [DAM]), *Cd45* (pan-hematopoietic), *Cd68* and *F4/80* (macrophages), and *Cd14* (monocytes and macrophages). We analyzed both the cortex and hippocampal areas. Although we analyzed fewer mice than in the histochemical analyses, we could still see that the level of *Gfap* mRNA was significantly increased by BCAS in *Btg2*^*−/−*^ mice (*p* = 0.0484, vs. sham-treated wild-type mice; *p* = 0.0362, vs. BCAS-treated wild-type mice; *p* = 0.0406, vs. sham*-treated Btg2*^*−/−*^ mice; Dunnett’s multiple comparison test). *Cd45* mRNA was also significantly higher in BCAS-treated *Btg2*^*−/−*^ mice than in sham-treated wild-type mice (*p* = 0.0490). On the other hand, mRNA levels of *Cd11b*, *Trem2*, *Dap12*, *Cd68*, *F4/80*, and *Cd14* (*p* = 0.1606, *p* = 0.340, *p* = 0.307, *p* = 0.1634, *p* = 0.7505, and *p* = 0.0923, respectively; one-way ANOVA) exhibited no significant difference, although BCAS-treated *Btg2*^*−/−*^ mice generally expressed higher levels of these mRNAs than the other groups (Fig. 7).

We also analyzed the protein levels of these glial cell markers in the same area, using ELISAs to confirm real-time PCR results. Although no significant difference was observed, likely due to the small number of mice examined, the level of GFAP tended to be higher in the BCAS-treated *Btg2*^*−/−*^ mice than in other groups, consistent with the real-time PCR results. The level of the microglial marker CD11b did not significantly differ among groups (Supplementary Fig. 4).

### Glial cell proliferation in *Btg2*^*−/−*^ mice in response to inflammatory stimuli

**Fig. 8:**
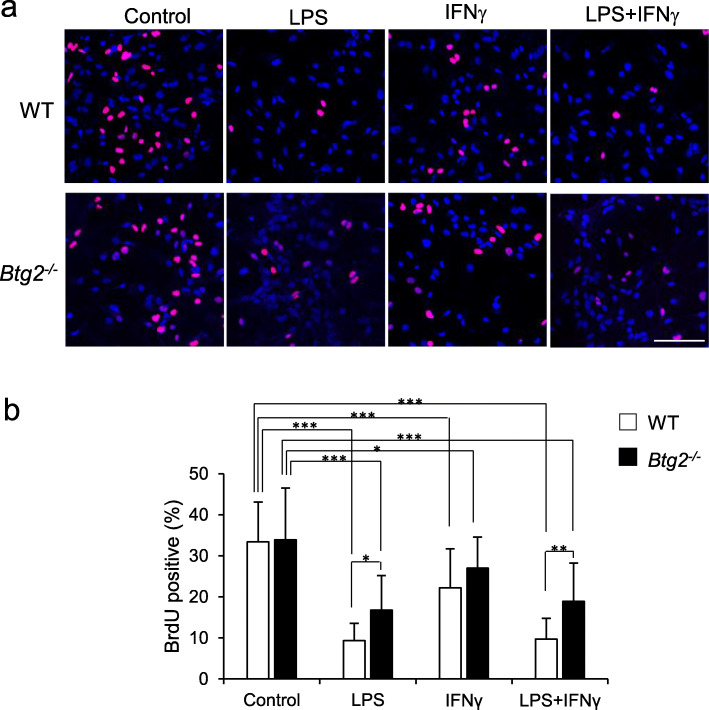
Cell proliferation analysis using 5-bromo-2′-deoxyuridine (BrdU) in mixed glial cells derived from wild-type and *Btg2*^*−/−*^ mice. **a** Images of BrdU-positive cells (red) and DAPI-positive total cells (blue) treated with vehicle control, 100 ng/ml LPS, 10 U/ml IFNγ, or 100 ng/ml LPS + 10 U /ml IFNγ. Scale bar = 100 μm. **b** BrdU-positive cells, as percentages of total DAPI-positive cells, were compared among groups. Means ± SD. *p < 0.05, **p < 0.01, and ***p < 0.001, as determined by Tukey–Kramer test

The results obtained with BCAS models suggested that *Btg2* deletion could increase the proliferation of glial cells during cerebral hypoperfusion. To further confirm the effects of *Btg2* deletion on glial cell proliferation in vitro, we performed BrdU incorporation assays in mixed glial cells derived from wild-type and *Btg2*^*−/−*^ mice. To mimic the pro-inflammatory conditions during cerebral hypoperfusion, mixed glial cells were treated with or without inflammatory stimuli (LPS and IFNγ), as reported [43] (Fig. 8). Cell proliferation rate was calculated as the ratio of BrdU-incorporating cells to total cells stained with DAPI. The ratio of BrdU-positive cells to total cells was significantly lower in the LPS, IFNγ, and LPS + IFNγ groups than in the control group. This result is consistent with a previous observation that inflammatory stimuli like LPS and IFNγ induce differentiation of proliferating primary glial cells into activated glial cells [44]. Importantly, the ratio of BrdU-positive cells to total cells was significantly higher in *Btg2*^*−/−*^ glial cells than in wild-type glial cells following LPS and LPS + IFNγ treatment, but no significant difference was observed after treatment with IFNγ alone (Fig. 8a, b). Co-immunostaining of the mixed glial cells with anti-BrdU and either anti-GFAP or anti-Iba1 antibody revealed that *Btg2* deletion altered responses to inflammatory stimuli in astrocytes, but not in microglia (Supplementary Fig. 5).

## Discussion

Although BTG2 is an anti-proliferative protein, and its expression is increased after ischemic stress, it has not been clearly determined whether deletion of this protein has impacts on the increase in glial cell number after cerebral hypoperfusion. In this study, we showed that deletion of *Btg2* increased the abundances of astrocytes and Mac2-positive cells in the white matter of optic tracts after BCAS, even though the severities of white matter lesions did not differ significantly between wild-type and *Btg2*^*−/−*^ mice.

Previous studies observed no difference between sham-operated and BCAS-treated wild-type mice in locomotor activity (as determined by the open-field test) or spatial learning ability (as determined by the Morris water maze) [5, 20]. Our findings were consistent with those studies. In addition, we observed behavioral changes in BCAS-treated *Btg2*^*−/−*^ mice relative to BCAS-treated wild-type mice: their locomotor activity increased in the open-field test, and their spatial learning ability was weak in the Morris water maze test. The ratio of travel distance in the outer or inner part of the open field to the total field did not significantly differ between wild-type and *Btg2*^*−/−*^ after BCAS, suggesting that the increase in locomotor activity in BCAS-treated *Btg2*^*−/−*^ mice was not related to anxiety or exploratory behavior. Because BTG2 is an anti-proliferative protein that is highly expressed in glial cells, these behavioral differences might be due to alteration of these cells caused by *Btg2* deletion.

BCAS treatment worsened white matter lesions and increased GFAP immunoreactivities irrespective of *Btg2* deletion, consistent with previous reports [4]. Moreover, immunoreactivities of GFAP and Mac2 were significantly higher in BCAS-treated *Btg2*^*−/−*^ mice than in BCAS-treated wild-type mice (Fig. 5), despite the lack of a difference in the white matter lesions (Fig. 4). Consistent with this, *Gfap* mRNA levels were higher in BCAS-treated *Btg2*^*−/−*^ mice than in sham-treated *Btg2*^*−/−*^ mice, whereas we observed no difference between wild-type mice with or without BCAS (Fig. 7). Brain ischemia induces the upregulation of *Gfap* mRNA levels for several weeks after the ischemic stress [45, 46]. Although *Gfap* mRNA levels were comparable between sham-treated and BCAS-treated wild-type mice 7 weeks after the surgery, the *Gfap* mRNA level in BCAS-treated *Btg2*^*−/−*^ mice might still be elevated at this time point. These results suggest that BTG2 negatively regulates astrocyte proliferation.

Mac2, also called galectin-3, was first reported as a macrophage-specific antigen defined by monoclonal antibodies [47]. It is expressed not only in activated microglia and macrophage/monocyte but also in astrocytes [48]. We examined the co-localization of Mac2 and GFAP or Iba1 in the white matter of BCAS-treated *Btg2*^*−/−*^ mice and observed that most of the Mac2-positive cells were not GFAP- or Iba1-positive. Therefore, we consider that the Mac2-positive cells that become more abundant after BCAS treatment are likely to be monocytes/macrophages. Although *Gfap* mRNA levels were significantly elevated, mRNA levels of markers of microglia and macrophage/monocytes exhibited no significant differences, except in the case of *Cd45*, a pan-hematopoietic marker. Further characterization of the increased abundance of Mac2-positive cells in BCAS-treated *Btg2*^*−/−*^ mice is necessary.

To determine whether BTG2 negatively regulates glial cell proliferation in response to inflammatory stimuli, we performed in vitro experiments using mixed glial cells from wild-type and *Btg2*^*−/−*^ mice. Incorporation of BrdU in response to inflammatory stimuli associated with brain ischemic conditions was greater in mixed glial cells from *Btg2*^*−/−*^ mice than in those from wild-type mice [49–51]. This result is consistent with multiple studies reporting that BTG2 plays an anti-proliferative role in breast cancer [52, 53], pancreatic cancer [54], and vertebral patterning by modulating bone morphogenesis [55], homeotic transformation of the axial skeleton [56, 57], and stress response [18]. In addition, our study shows for the first time that BTG2 regulates astrocyte proliferation in chronic cerebral hypoperfusion models.

This study had several limitations. First, we examined BrdU incorporation in vitro using mixed glial cells, but not in separated astrocytes or microglia. By co-immunostaining mixed glial cells using both anti-BrdU and anti-GFAP or anti-Iba1 antibody, we observed that responses to inflammatory stimuli were altered by *Btg2* deletion in astrocytes, but not in microglia. However, it remains unclear whether these effects in astrocytes with *Btg2* deletion are cell-autonomous or non-cell-autonomous. This in vitro culture system also did not include macrophages/monocytes, although it is possible that the Mac2-positive cells we observed were macrophages/monocytes. In a future study, we will examine the effect of BTG2 on the proliferation of each cell type using conditional cell type–specific deletions of *Btg2*. Second, we could not determine why the increased abundance of astrocytes and Mac2-positive cells in BCAS-treated *Btg2*^*−/−*^ mice vs. BCAS-treated wild-type mice was observed in the optic tract but not the corpus callosum. This observation might be related to regional differences in vulnerabilities to ischemic stress or that of the expression level of *Btg2*. Indeed, previous studies have reported regional differences in the distribution of neuropeptides [58] and nitric oxide synthase [59] in cerebral blood vessels. Finally, although we have tried several anti-BTG2 antibodies, including commercial ones as well as one developed in our laboratory, none of them worked well in western blotting or immunohistochemistry of samples from wild-type and *Btg2*^*−/−*^ mice. Further studies using BTG2-specific antibodies are required to fully characterize when and where BTG2 functions to regulate glial cell proliferation during chronic cerebral hypoperfusion.

## Conclusions

Deletion of *Btg2* caused behavioral changes associated with altered responses of glial cells, especially those of astrocytes and Mac2-positive cells. Moreover, our in vitro experiments supported the idea that BTG2 negatively regulates proliferation of glial cells. These results suggest that BTG2 might serve as an anti-proliferative protein in glial cells during cerebral hypoperfusion, thereby protecting against vascular dementia.

## Supplementary Information

**Additional file 1: Supplementary Figure 1.** Anxiety/exploratory parameters in the open-field test. The ratio of distance travelled in the inner zone (a) or outer zone (b) to the distance traveled overall was compared among groups by Tukey’s HSD test. Data are adjusted means ± SEM. n.s., not significant. **Supplementary Figure 2.** Probe test results of the Morris water maze test. NE: northeast quadrant area; NW: northwest; SE: southeast; SW: southwest. The platform was in the NE quadrant area (target quadrant). Time spent in each non-target quadrant was compared with the duration spent in the target quadrant in each group. Data are adjusted means ± SEM. ***p < 0001; Tukey–Kramer test. The following numbers of mice were used: wild-type + Sham, n = 13; wild-type + BCAS, n = 15; *Btg2*^*-/-*^ + Sham, n = 11; *Btg2*^*-/-*^ + BCAS, n = 16. **Supplementary Figure 3.** Immunohistochemical observations of glia cells in the corpus callosum (CC) of sham/BCAS-treated wild-type and *Btg2*^*-/-*^ mice. (a) Staining for GFAP (upper), Iba1 (middle), and Mac2 (lower). Scale bar = 20μm. CC is inside the white dotted lines. (b) Immunoreactive (IR) areas were compared among groups after adjusting for sex. Adjusted mean ± SEM. **p < 0.01 and ***p < 0.001, as determined by Tukey’s HSD test. n.s., not significant. **Supplementary Figure 4.** Protein levels of astrocyte marker GFAP (a) and microglial marker CD11b (b) in the brain of sham/BCAS-treated wild-type and *Btg2*^*-/-*^ mice. All mice were male. Data are means ± SD. Differences were evaluated by Dunnett’s multiple comparison test. n.s., not significant. **Supplementary Figure 5.** Cell proliferation analysis using 5-bromo-2’-deoxyuridine (BrdU) in mixed glial cells derived from wild-type and *Btg2*^*-/-*^ mice. Samples were co-immunostained with anti-GFAP (a and c) or anti-Iba1 antibodies (b and d). Images show BrdU-positive (red), DAPI-positive (blue), and GFAP-positive cells (green, a) or Iba1-positive cells (green, b) treated with vehicle control, 100 ng/ml LPS, 10 U/ml IFNγ, or 100 ng/ml LPS + 10 U/ml IFNγ. The proportion of double-positive cells for BrdU and GFAP (c) or Iba1 (d), as percentages of total GFAP (c) or Iba1 (d) -positive cells, was compared among groups. Means ± SD. *p < 0.05, and ***p < 0.001, as determined by Student’s *t*-test. Scale bar = 100 μm. **Supplementary Table 1.** Two- or three-way ANOVA Results

## Data Availability

All data generated or analyzed during this study are available upon reasonable request.

## Abbreviations

BTG2: B-cell translocation gene-2
BCAS: Bilateral common carotid artery stenosis
GFAP: Glial fibrillary acidic protein
Iba1: Ionized calcium binding adapter molecule-1
