# Supplementary material for: Deletion of B-cell translocation gene 2 (BTG2) alters the responses of glial cells in white matter to chronic cerebral hypoperfusion
Source: J Neuroinflammation. 2021 Apr 3;18:86. doi: 10.1186/s12974-021-02135-w (PMC8019185; doi:10.1186/s12974-021-02135-w)
Supplement: Supplementary file 1 — Additional file 1: Supplementary Figure 1. Anxiety/exploratory parameters in the open-field test. The ratio of distance travelled in the inner zone (a) or outer zone (b) to the distance traveled overall was compared among groups by Tukey’s HSD test. Data are adjusted means ± SEM. n.s., not significant. Supplementary Figure 2. Probe test results of the Morris water maze test. NE: northeast quadrant area; NW: northwest; SE: southeast; SW: southwest. The platform was in the NE quadrant area (target quadrant). Time spent in each non-target quadrant was compared with the duration spent in the target quadrant in each group. Data are adjusted means ± SEM. ***p < 0001; Tukey–Kramer test. The following numbers of mice were used: wild-type + Sham, n = 13; wild-type + BCAS, n = 15; Btg2-/- + Sham, n = 11; Btg2-/- + BCAS, n = 16. Supplementary Figure 3. Immunohistochemical observations of glia cells in the corpus callosum (CC) of sham/BCAS-treated wild-type and Btg2-/- mice. (a) Staining for GFAP (upper), Iba1 (middle), and Mac2 (lower). Scale bar = 20μm. CC is inside the white dotted lines. (b) Immunoreactive (IR) areas were compared among groups after adjusting for sex. Adjusted mean ± SEM. **p < 0.01 and ***p < 0.001, as determined by Tukey’s HSD test. n.s., not significant. Supplementary Figure 4. Protein levels of astrocyte marker GFAP (a) and microglial marker CD11b (b) in the brain of sham/BCAS-treated wild-type and Btg2-/- mice. All mice were male. Data are means ± SD. Differences were evaluated by Dunnett’s multiple comparison test. n.s., not significant. Supplementary Figure 5. Cell proliferation analysis using 5-bromo-2’-deoxyuridine (BrdU) in mixed glial cells derived from wild-type and Btg2-/- mice. Samples were co-immunostained with anti-GFAP (a and c) or anti-Iba1 antibodies (b and d). Images show BrdU-positive (red), DAPI-positive (blue), and GFAP-positive cells (green, a) or Iba1-positive cells (green, b) treated with vehicle control, 100 ng/ml LPS, 10 U/ml IFN [file 12974_2021_2135_MOESM1_ESM.docx]

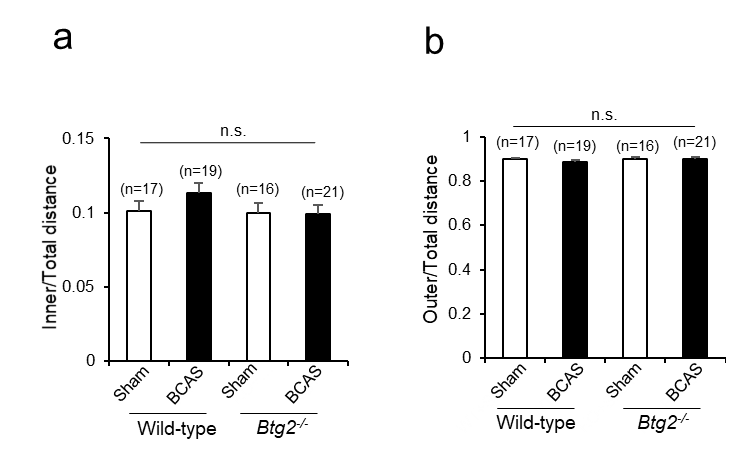


**Supplementary Figure 1.** Anxiety/exploratory parameters in the open-field test. The ratio of distance travelled in the inner zone (a) or outer zone (b) to the distance traveled overall was compared among groups by Tukey’s HSD test. Data are adjusted means ± SEM. n.s., not significant.


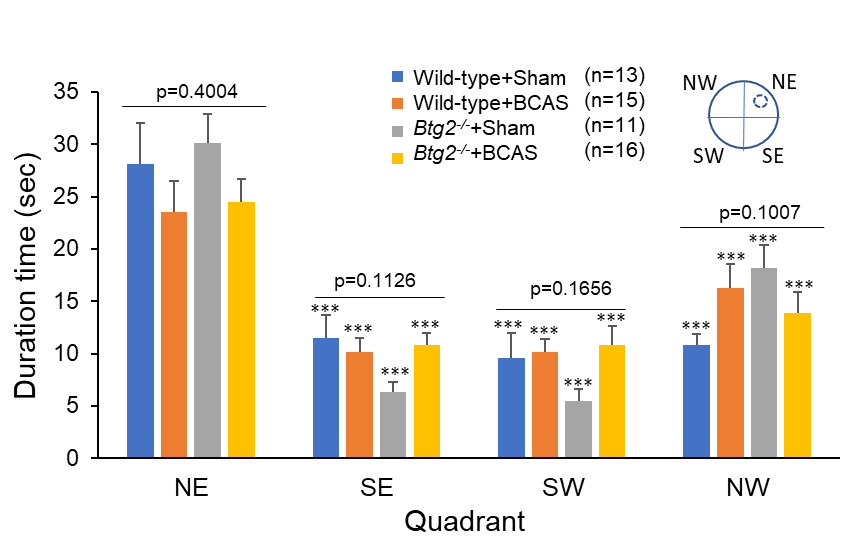


**Supplementary Figure 2.** Probe test results of the Morris water maze test. NE: northeast quadrant area; NW: northwest; SE: southeast; SW: southwest. The platform was in the NE quadrant area (target quadrant). Time spent in each non-target quadrant was compared with the duration spent in the target quadrant in each group. Data are adjusted means ± SEM. ****p* < 0001; Tukey–Kramer test. The following numbers of mice were used: wild-type + Sham, n = 13; wild-type + BCAS, n = 15; *Btg2^-/-^* + Sham, n = 11; *Btg2^-/-^* + BCAS, n = 16.

**
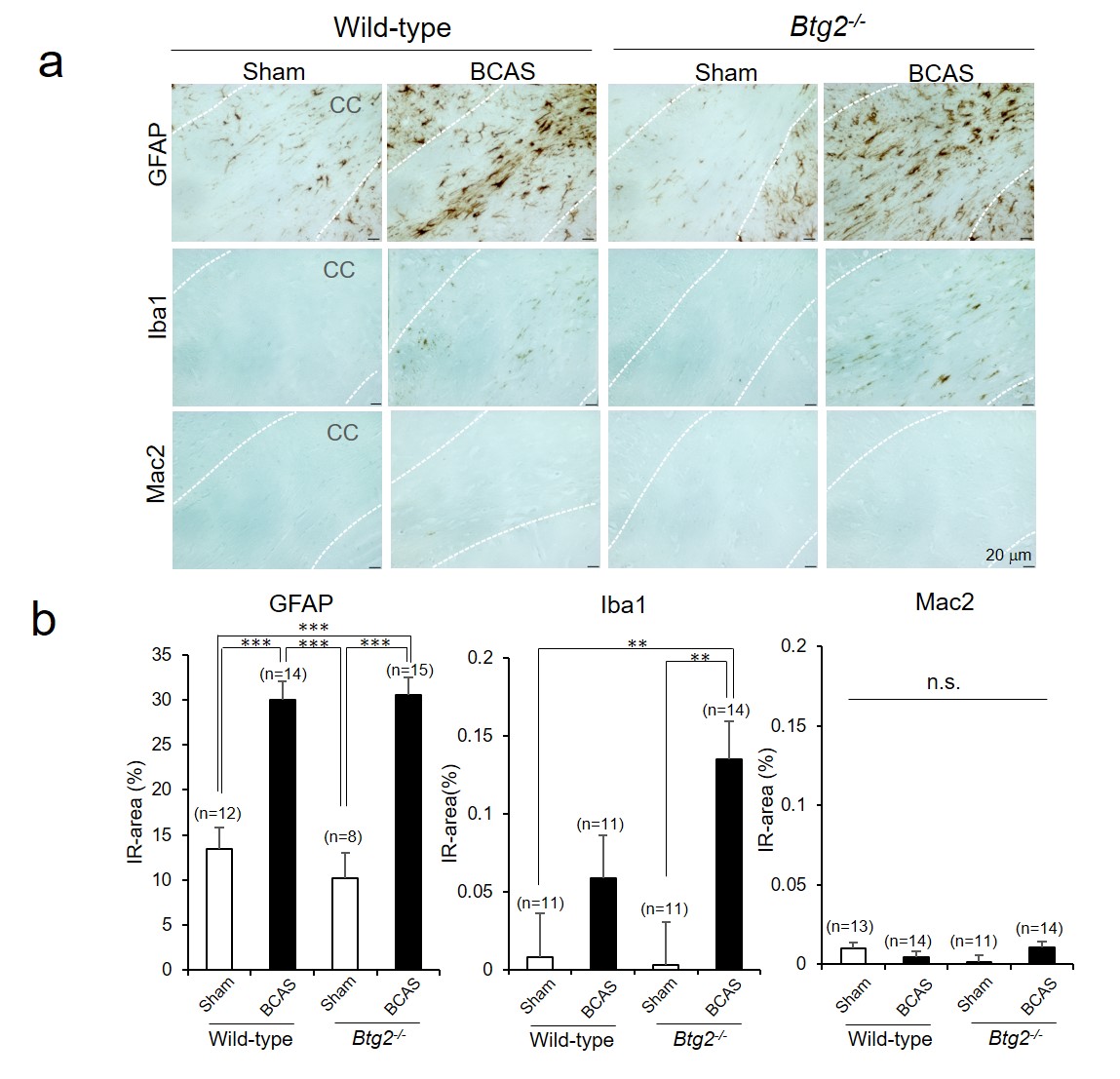
Supplementary Figure 3.** Immunohistochemical observations of glia cells in the corpus callosum (CC) of sham/BCAS-treated wild-type and *Btg2^-/-^* mice. (a) Staining for GFAP (upper), Iba1 (middle), and Mac2 (lower). Scale bar = 20 μm. CC is inside the white dotted lines. (b) Immunoreactive (IR) areas were compared among groups after adjusting for sex. Adjusted mean ± SEM. ***p* < 0.01 and ****p* < 0.001, as determined by Tukey’s HSD test. n.s., not significant.


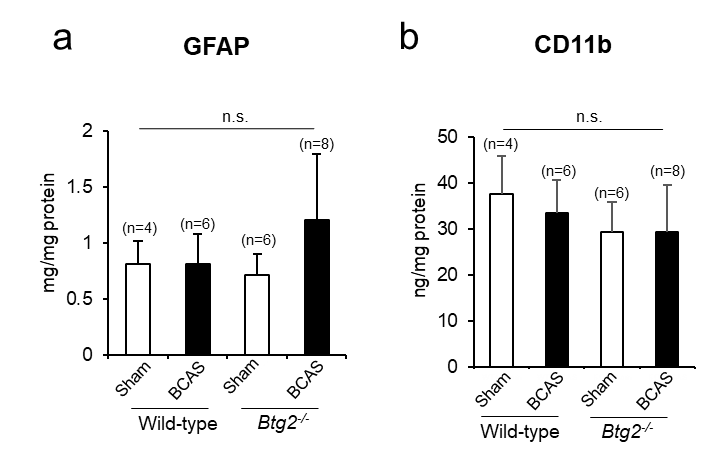


**Supplementary Figure 4.** Protein levels of astrocyte marker GFAP (a) and microglial marker CD11b (b) in the brain of sham/BCAS-treated wild-type and *Btg2^-/-^* mice. All mice were male. Data are means ± SD. Differences were evaluated by Dunnett’s multiple comparison test. n.s., not significant.


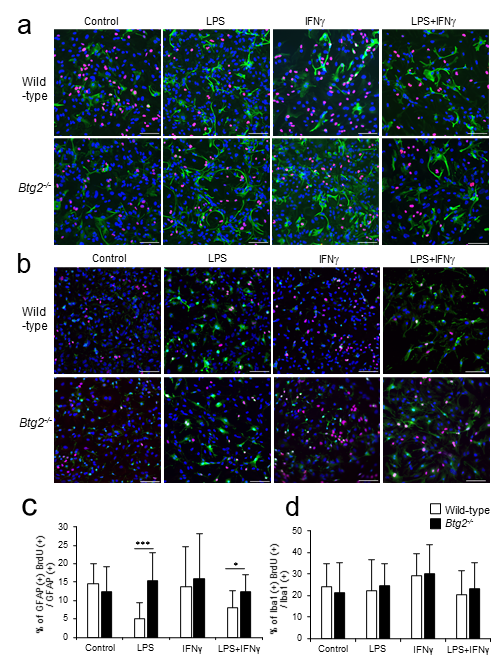


**Supplementary Figure 5.** Cell proliferation analysis using 5-bromo-2’-deoxyuridine (BrdU) in mixed glial cells derived from wild-type and *Btg2^-/-^* mice. Samples were co-immunostained with anti-GFAP (a and c) or anti-Iba1 antibodies (b and d). Images show BrdU-positive (red), DAPI-positive (blue), and GFAP-positive cells (green, a) or Iba1-positive cells (green, b) treated with vehicle control, 100 ng/ml LPS, 10 U/ml IFNγ, or 100 ng/ml LPS + 10 U/ml IFNγ. The proportion of double-positive cells for BrdU and GFAP (c) or Iba1 (d), as percentages of total GFAP (c) or Iba1 (d) -positive cells, was compared among groups. Means ± SD. **p* < 0.05, and ****p* < 0.001, as determined by Student’s *t*-test. Scale bar = 100 μm.

**Supplementary Table 1 Two- or three-way ANOVA Results**

**<Behavioral test>**

**Open Field Test**

Total Distance (Fig.3a)

| Comparisons | Estimate | p-value |
| --- | --- | --- |
| Sham vs. BCAS | 1.5182103 | 0.1955 |
| WT vs. KO | 3.9461392 | 0.0011 |
| Male vs. Female | 1.4232961 | 0.2341 |

Average Speed (Fig.3b)

| Comparisons | Estimate | p-value |
| --- | --- | --- |
| Sham vs. BCAS | 0.0016997 | 0.1941 |
| WT vs. KO | 0.0043570 | 0.0013 |
| Male vs. Female | 0.0016094 | 0.2280 |

　Inner/Total Distance (Fig.S1a)

| Comparisons | Estimate | p-value |
| --- | --- | --- |
| Sham vs. BCAS | 0.0035616 | 0.2767 |
| WT vs. KO | -0.0036910 | 0.2592 |
| Male vs. Female | 0.0044563 | 0.1835 |

　Outer/Total Distance (Fig.S1b)

| Comparisons | Estimate | p-value |
| --- | --- | --- |
| Sham vs. BCAS | -0.003559 | 0.2770 |
| WT vs. KO | 0.0036887 | 0.2595 |
| Male vs. Female | -0.004454 | 0.1838 |

**Morris Water Maze Test**

　Escape Latency (Fig.3c)

| Comparisons | Estimate | p-value |
| --- | --- | --- |
| Sham vs. BCAS | 2.2318831 | 0.1177 |
| WT vs. KO | -1.601138 | 0.2624 |
| Male vs. Female | -1.296189 | 0.3753 |
| Day5 vs. Day1 | 14.658643 | <.0001 |
| Day5 vs. Day2 | 9.8028393 | 0.0005 |
| Day5 vs. Day3 | -3.3360 | 0.2345 |
| Day5 vs. Day4 | -6.196268 | 0.0277 |

Probe test: Source in NE quadrant (Fig.S2)

| Comparisons | Estimate | p-value |
| --- | --- | --- |
| Sham vs. BCAS | -2.61827 | 0.0880 |
| WT vs. KO | 0.36732 | 0.8108 |
| Male vs. Female | -2.20740 | 0.1542 |

SE quadrant (Fig.S2)

| Comparisons | Estimate | p-value |
| --- | --- | --- |
| Sham vs. BCAS | 0.792141 | 0.2991 |
| WT vs. KO | -1.164615 | 0.1346 |
| Male vs. Female | -0.238117 | 0.7570 |

NW quadrant (Fig.S2)

| Comparisons | Estimate | p-value |
| --- | --- | --- |
| Sham vs. BCAS | 0.3294519 | 0.7474 |
| WT vs. KO | 1.5093323 | 0.1498 |
| Male vs. Female | 1.5554277 | 0.1378 |

SW quadrant (Fig.S2)

| Comparisons | Estimate | p-value |
| --- | --- | --- |
| Sham vs. BCAS | 1.5048799 | 0.0870 |
| WT vs. KO | -0.707109 | 0.4226 |
| Male vs. Female | 0.8821577 | 0.3177 |

**<Histology>**

**White Matter Lesion**

Optic tract (Fig.4a)

| Comparisons | Estimate | p-value |
| --- | --- | --- |
| Sham vs. BCAS | 0.4691455 | <.0001 |
| WT vs. KO | 0.0008848 | 0.9879 |
| Male vs. Female | -0.080992 | 0.1663 |

Corpus Callosum (Fig.4b)

| Comparisons | Estimate | p-value |
| --- | --- | --- |
| Sham vs. BCAS | 0.3894748 | <.0001 |
| WT vs. KO | 0.0144247 | 0.7862 |
| Male vs. Female | -0.069695 | 0.1869 |

**IHC in Optic Tract**

Anti-GFAP (Fig.5b)

| Comparisons | Estimate | p-value |
| --- | --- | --- |
| Sham vs. BCAS | 13.704755 | <.0001 |
| WT vs. KO | 4.7916824 | 0.0326 |
| Male vs. Female | -2.57334 | 0.2444 |

Anti-Iba1 (Fig.5b)

| Comparisons | Estimate | p-value |
| --- | --- | --- |
| Sham vs. BCAS | 0.4479716 | 0.0033 |
| WT vs. KO | 0.2709234 | 0.0795 |
| Male vs. Female | 0.315303 | 0.0399 |

Anti-Mac2 (Fig.5c)

| Comparisons | Estimate | p-value |
| --- | --- | --- |
| Sham vs. BCAS | 0.4706137 | 0.0016 |
| WT vs. KO | 0.2854907 | 0.0590 |
| Male vs. Female | -0.136201 | 0.3607 |

**IHC in Corpus Callosum**

Anti-GFAP (Fig.S3b)

| Comparisons | Estimate | p-value |
| --- | --- | --- |
| Sham vs. BCAS | 9.410818 | <.0001 |
| WT vs. KO | -1.346834 | 0.2606 |
| Male vs. Female | -2.114925 | 0.0755 |

Anti-Iba1 (Fig.S3b)

| Comparisons | Estimate | p-value |
| --- | --- | --- |
| Sham vs. BCAS | 0.0421116 | 0.0027 |
| WT vs. KO | 0.0191664 | 0.1813 |
| Male vs. Female | 0.0083793 | 0.5543 |

Anti-Mac2 (Fig.S3b)

| Comparisons | Estimate | p-value |
| --- | --- | --- |
| Sham vs. BCAS | 0.0002347 | 0.8990 |
| WT vs. KO | -0.000909 | 0.6336 |
| Male vs. Female | -0.001094 | 0.5610 |

**<Realtime PCR>**

*Gfap* (Fig.7a)

| Comparisons | Estimate | p-value |
| --- | --- | --- |
| Sham vs. BCAS | 0.1648222 | 0.087 |
| WT vs. KO | 0.1711533 | 0.0763 |

*Cd11b* (Fig.7b)

| Comparisons | Estimate | p-value |
| --- | --- | --- |
| Sham vs. BCAS | 0.0008172 | 0.6592 |
| WT vs. KO | 0.0042122 | 0.0319 |

*Trem2* (Fig.7c)

| Comparisons | Estimate | p-value |
| --- | --- | --- |
| Sham vs. BCAS | 0.0090913 | 0.3256 |
| WT vs. KO | 0.0090333 | 0.3286 |

*Dap12* (Fig.7d)

| Comparisons | Estimate | p-value |
| --- | --- | --- |
| Sham vs. BCAS | 0.0515274 | 0.1194 |
| WT vs. KO | 0.0290153 | 0.3706 |

*Cd45* (Fig.7e)

| Comparisons | Estimate | p-value |
| --- | --- | --- |
| Sham vs. BCAS | 0.0017257 | 0.0482 |
| WT vs. KO | 0.0012755 | 0.1355 |

*Cd68* (Fig.7f)

| Comparisons | Estimate | p-value |
| --- | --- | --- |
| Sham vs. BCAS | 0.0038101 | 0.1955 |
| WT vs. KO | 0.0041214 | 0.1629 |

*F4/80* (Fig.7g)

| Comparisons | Estimate | p-value |
| --- | --- | --- |
| Sham vs. BCAS | 0.000702 | 0.6587 |
| WT vs. KO | 0.0006214 | 0.6957 |

*Cd14* (Fig. 7h)

| Comparisons | Estimate | p-value |
| --- | --- | --- |
| Sham vs. BCAS | 0.0010818 | 0.4595 |
| WT vs. KO | 0.0035923 | 0.0210 |

**<ELISA>**

GFAP (Fig.S4a)

| Comparisons | Estimate | p-value |
| --- | --- | --- |
| Sham vs. BCAS | 0.1230361 | 0.1564 |
| WT vs. KO | 0.0737407 | 0.3879 |

CD11b (Fig.S4b)

| Comparisons | Estimate | p-value |
| --- | --- | --- |
| Sham vs. BCAS | -1.007814 | 0.5713 |
| WT vs. KO | -3.118482 | 0.0901 |
